# A Review of Features in Internet Consumer Health Decision-support Tools

**DOI:** 10.2196/jmir.4.2.e11

**Published:** 2002-11-22

**Authors:** Gary Schwitzer

**Affiliations:** ^1^University of MinnesotaMinnesotaUSA

**Keywords:** decision making, informatics, Internet, multimedia, social support, treatment outcome, prognosis

## Abstract

**Background:**

Over the past decade, health care consumers have begun to benefit from new Web-based communications tools to guide decision making on treatments and tests. Using today's online tools, consumers who have Internet connections can: watch and listen to videos of physicians; watch and hear the stories of other consumers who have faced the same decisions; join an online social support network; receive estimates of their own chances of experiencing various outcomes; and do it all at home.

**Objective:**

To review currently-available Internet consumer health decision-support tools.

**Methods:**

Five Web sites offering consumer health decision-support tools are analyzed for their use of 4 key Web-enabled features: the presentation of outcomes probability data tailored to the individual user; the use of videotaped patient interviews in the final product to convey the experiences of people who have faced similar diagnoses in the past; the ability to interact with others in a social support network; and the accessibility of the tool to any health care consumers with an Internet connection.

**Results:**

None of the 5 Web sites delivers all 4 target features to all Web users. The reasons for these variations in the use of key Web functionality — features that make the Web distinctive — are not immediately clear.

**Conclusions:**

Consumers trying to make health care decisions may benefit from current Web-based decision-support tools. But, variations in Web developers' use of 4 key Web-enabled features leaves the online decision-support experience less than what it could be. Key research questions are identified that could help in the development of new hybrid patient decision-support tools.

## Introduction

### A Decade of Development of Decision-support Tools

If, 10 years ago, health communicators had learned that they could deliver programs to peoples' homes on demand, with video, data tailored to the individual user, and with a social support network in place through these programs, many may have jumped at the opportunity. Today all of that can be done through the use of the Internet. However, while it is possible that this research overlooked a new Web site, I did not find any current health-related Web site that offers consumer health decision-support tools using all of the Web features described.

Since the early 1990s, there has been an evolution in development of patient-targeted decision-support tools in different media: printed materials, videotapes, CD-ROMs, computer-based interactive multimedia (laserdisc) programs, and Web-based programs [1]. A 1997 report by the Agency for Health Care Policy and Research stated: "Although patient health informatics tools can potentially empower patients to make more informed choices, there is limited empirical evidence of the outcomes of their use and of their overall value." [2] Since then, reviews of trials of decision aids [3] and of the potential impact of such tools on clinical practice [4] have been published. Meager though such outcomes evaluation may be, it may nonetheless outpace the formative and process evaluation in the development of such tools. Perhaps because many of the development efforts are conducted behind the walls of proprietary ventures, there is little public information available on the development of Internet consumer health decision-support tools. This review analyzes 4 areas of variability in the use of key Web-enabled features in online decision-support tool development. The features in question are some of the key functionalities that distinguish the Web from other media. No other medium can deliver the combination of these 4 features.

### Web-enabled Features

1. **Patient video interviews.** Some developers choose to deliver multimedia patient stories while others do not. In Internet trade publications, there are anecdotal reports from Web developers about how multimedia narratives help people relate to the content [5]. In the academic setting, the Institute for New Media Studies at the University of Minnesota is developing a "best practices" list of online storytelling features and methods to guide Web content developers [6]. The Institute's analysis demonstrates the variations in Web design and content delivery styles, and reminds us, "The Web must go through a maturation process; the same process all new media have undergone." A Pew Internet & American Life Project report estimates that 21% of American Internet users have high-speed connections at home — the type of connection required for best use of multimedia [7]. Nearly half of those users say the Internet has "improved the way they get health care information." And, nearly half report using some kind of multimedia content during a typical day online. But I am not aware of any current information on multimedia use by health information seekers.
2. **Online community network.** Some developers offer online social support while others do not. The popularity of discussion groups or online "communities" is reflected in the traffic figures reported by some Web sites [8]. Computer-industry trade publications reflected on the popularity of online communities among health information seekers as long as 6 years ago [9]. While general health information seekers have shown some reluctance to join online health communities, there is evidence that those in poorer health are more inclined to use these features [10]. In one randomized clinical trial, online community members reported feeling better while lowering health care costs [11].
3. **User-specific outcomes data.**
**Some** Web sites emphasize tailored prognostic data to users while others do not. Tailored prognostic data was a key component of one of the pioneering development efforts in the field of shared decision-making and decision support more than a decade ago [12]. Yet, until recently (until introduced as a primary feature of one of the new products in this field today [13]), the offering of user-specific outcomes data has remained on the sidelines of Internet decision-support tool development.
4. **Free, public access.** Some Web sites offer such tools to the general public at no cost and with no requirement that the user register while others require subscriptions, licenses, or registration. For newly-diagnosed individuals, the ability to use the Internet for free to get immediate access to information at any time of day or night has always been an attractive feature.

## Methods

I chose Web sites for review based on the following criteria:

- The site must offer consumers detailed information on health care treatment options and potential outcomes to help them in their decision-making.
- The site must offer such information for several different health care treatment decisions. Numerous Web sites address only one topic or condition but these were excluded from this review.
- The products must be developed for the Internet. Products originally developed for print and then converted with little or no change for Web use were excluded, since the features being reviewed were special features of the Internet. One noteworthy Web site that was excluded from review is that of the Ottawa Health Research Institute [14]. The site offers portable document format (PDF) files on 17 different decision topics. But, each is described as a booklet, worksheet, or workbook and was not designed primarily for delivery on the Internet. Since the decision aids posted there were adaptations from print or audiotape products, they were excluded from review.

The MEDLINE and Cochrane Library databases (Cochrane Database of Systematic Reviews) were searched. Individuals who are active in the field were contacted for information on current Internet decision-support tools.

## Results

Five Web sites were analyzed:

- The Comprehensive Health Enhancement Support System (CHESS)
- Database of Individual Patient Experiences (DIPEx)
- Foundation for Informed Medical Decision Making (FIMDM) in partnership with HealthDialog, Inc
- MayoClinic.com
- NexCura, Inc

### Comparison of Features in Web-based Patient Decision-making Tools

After a decade of development, there is still no Web site that offers all of the features analyzed in this paper: patient video interviews, an online community network, user-specific outcomes data, and free, public access. Table 1 presents a chart comparing the features of these Internet consumer health decision-making tools.

**Table 1:** Comparison of features in Internet patient decision-support tools

	Patient video interviews	Online community network	User-specific outcomes data	Access
FIMDM*	No	No	No	Web services for subscribers only
CHESS	Yes	Yes	No	Access limited to consortium member groups
NexCura	No	No	Yes	Free access through co-branded partner site; registration required
DIPEx	Yes	No†	No	Free on the Web
MayoClinic.com	Yes	No	No‡	Free on the Web

^*^ Currently none of FIMDM's outcomes data and patient interviews are available on the Internet.

^†^ DIPEx is currently redesigning and evaluating the concept of an online social support network.

^‡^ MayoClinic.com pilot project to tailor breast cancer adjuvant therapy data is in development.

### Dartmouth/FIMDM/Health Dialog Shared Decision-making Programs

In the late 1980s, a team of researchers based at Dartmouth Medical School began an experiment in communicating with newly-diagnosed individuals in new ways. Beginning with the topic of benign prostatic hyperplasia (BPH), the Dartmouth-based group began to communicate with health care consumers about the risks and benefits of alternative treatment and testing options for given conditions.

The team (which became the not-for-profit entity named the Foundation for Informed Medical Decision Making or FIMDM) adopted a rigorous development and review process for the content of these "shared decision-making programs" [12]. There were several distinguishing features. First, the programs' outlines of risks and benefits of alternative treatment options were based on outcomes research. At the core of the initial FIMDM development effort was the federally-funded Prostate Disease Patient Outcomes Research Team [15]. Second, the team chose to deliver the program content on a computer-based platform. This decision was based primarily on the researchers' interest in offering the individual the chance to learn his own chances of experiencing the various outcomes being described in the program. The user was asked to provide some personal information (age, symptom severity, and general health description) at the beginning of the viewing session. The computer then pulled the available outcomes data stored in the computer's database to provide to each user his own chances of experiencing the various risks and benefits of a surgical or nonsurgical choice. Third, the team conducted interviews and focus groups with health care consumers at an early stage of the program development process. What was learned from consumers (and from people who had faced the diagnoses in question in the past) made a direct impact on the final product being developed. Fourth, the stories of others who faced these diagnoses, made different treatment choices, and had different outcomes and experiences were videotaped and used in the final program. The developers believed that these video interviews would convey the real human experiences described by others who had "been there already" — better than text alone, or any combination of text, still photographs, and audio. A laserdisc player was part of the original platform because the computer could easily access video clips from it and because it delivered high-quality, full-screen, full-motion video images.

#### Early Evidence of Impact

Two large managed care companies, in a pilot program using FIMDM's BPH program, reported that users of the program often chose a less-costly nonsurgical approach to the management of their urinary symptoms [16]. Most men had very positive reactions to the program, rating it generally clear, informative, balanced, and useful in helping them make a treatment decision [17]. These pilot studies provided FIMDM the impetus for creation of other shared decision-making programs. Studies of other programs produced by FIMDM attempted to document the impact of programs on prostate cancer screening [18], chronic low back pain [19,20], and ischemic heart disease [21].

At first, FIMDM worked with a business development partner (Sony Medical Systems) to license its programs to health care providers, health plans, and managed care companies. Consumers could only view the programs in the clinical setting if referred to them by their health care provider. FIMDM wanted to emphasize that these were shared decision-making programs, to be used by consumers and clinicians working together. Limiting access to the clinical setting helped ensure immediate follow-up with a physician or nurse to answer questions raised by the program. Nonetheless, providers struggled to integrate the programs into their clinical practice routine. The platform upon which the programs ran was functional (a computer, touch-screen monitor, laserdisc player, and printer). But, many viewed it as cumbersome, or as one observer described it, "expensive, unwieldy and difficult to access" [22]. The computer-and-laserdisc platform for these programs was abandoned in the mid-1990s. The programs were transferred to videotape, but in that transfer, the tailoring of the information to the individual was lost. The question of the value of such tailoring was never adequately studied.

#### FIMDM/Health Dialog today

Today, FIMDM and its business partner (Health Dialog, Inc, Boston) license to subscribers a telephonic nurse "health coach" service, multimedia decision-making modules on videotape and CD-ROM, a self-care handbook, and some related Web-based information [23]. However, none of the FIMDM/Health Dialog decision-support content is available for free to the general public on the Internet.

##### Features

1. **Patient video interviews.** Multimedia patient stories are a core part of the FIMDM products, but none is currently offered online.
2. **Online community network.** An online social support network with other patients or consumers has never been part of the FIMDM product package, although a telephonic nurse "health coach" service is now available to subscribers.
3. **User-specific outcomes data.** FIMDM once offered tailored prognostic data on interactive computer and laserdisc programs but this feature is not available on the Internet at this time.
4. **Free, public access.** All FIMDM programs are now offered only through licenses to subscriber organizations. None is available free online to the general public.

### CHESS

At about the same time in the late 1980s that the Dartmouth team was beginning work on its prototype computer-and-laserdisc program, a team with a somewhat similar mission was assembling programs at the University of Wisconsin in Madison. The Wisconsin project became known as Comprehensive Health Enhancement Support System or CHESS [24]. Its early programs addressed topics such as living with breast cancer, living with HIV/AIDS, adult children of alcoholics, stress management, and sexual assault. As is evident from some of the topics addressed, decision support is just one goal of the CHESS programs, and does not appear to be the primary goal. Part of the group's online mission statement is to develop "interactive health communication technologies that: . . . offer a variety of ways to access information, emotional support, and tools for decision making and health risk reduction [24]."

#### Community or social support

One distinguishing feature of the CHESS programs is the ability of users to exchange messages with others in online discussion groups or bulletin boards or submit "ask the expert" questions. The group has published results of its computer-based breast cancer social support network [26]. Among the support group benefits cited by women are these:

- "anonymity within the support group fostered equalized participation and allowed women to communicate in ways that would have been more difficult in a face-to-face context";
- "abundant emotional support, encouragement and informational support";
- the chance to "change their focus from a preoccupation with their own sickness to thinking of others."

#### CHESS changes through the years

For years, the CHESS programs suffered from some of the same access and delivery problems as the FIMDM computer-laserdisc programs. At first, CHESS used a DOS computer platform and loaned the systems to users. The platform changed from DOS to Windows and now programs are delivered on the Web — but not to the general public. Patients or employees of 9 member-organizations of the CHESS Health Education Consortium must register and use a password to access the programs [27]. Current CHESS modules address the following topics: breast cancer, prostate cancer, smoking cessation, heart disease, asthma, menopause, dementia, and care giving.

##### Features

1. **Patient video interviews.** Multimedia patient stories are offered in the CHESS modules.
2. **Online community network.** CHESS is the only Web-based decision-support tool reviewed that offers a community or social support feature.
3. **User-specific outcomes data.** CHESS modules do not include tailored prognostic data.
4. **Free, public access.** All CHESS modules are now offered only to patients or employees of 9 member-organizations of the CHESS Health Education Consortium who must register to gain access. No modules are available online to the general public.

### NexCura.com

In late 1999, Internet decision-support software began to be offered by a company now named NexCura, Inc [13]. The software is distributed through organizations that partner with NexCura, through so-called "co-branding" arrangements. These partners include the American Cancer Society, the American Heart Association, patient advocacy groups, payers, providers, and health care portal Web sites. The company claims to reach over 100000 registered patients with its oncology (Cancer Profiler™) topics alone. The programs require a user to provide diagnostic and test result information. The Profiler software matches that data with research studies and delivers information on treatment options and outcomes probabilities. Consumers can also see summaries of recent studies related to their condition. Treatment outcomes information is currently available for bladder, breast, colorectal, non-small cell lung, ovarian, prostate, and small cell lung cancers.

The presentation of evidence-based outcomes probabilities with this product is detailed, but requires a consumer to be comfortable with complicated presentation of material. An example of the co-branded Breast Cancer Profiler appears on the Web site of the Y-ME National Breast Cancer Organization [28]. Online registration is required.

#### Features

1. **Patient video interviews.** Multimedia patient stories are not offered in the NexCura tools.
2. **Online community network.** No community or social support feature is available in the NexCura tools.
3. **User-specific outcomes data.** NexCura tools emphasize the use of tailored prognostic data.
4. **Free, public access.** All NexCura tools are now offered free through co-branded partner Web sites. Registration is required.

### MayoClinic.com

One of the first consumer health Web sites offering information free to all users on the World Wide Web was one produced by the Mayo Clinic in 1995. The latest edition of that site is called MayoClinic.com, launched in late 2000. The site offers decision-support modules (called Health Decision Guides) on early-stage breast cancer [29], herniated disks [30], middle ear infections [31], anterior cruciate ligament knee injuries [32], colorectal cancer screening [33], and early-stage prostate cancer [34]. Others in development will address breast cancer adjuvant therapy, benign uterine conditions, and hormone replacement therapy. The programs are still available for free to anyone on the Web. Patients' stories about their treatment choice and their experience with those choices are delivered in text and in video (RealPlayer® plug-in required). There are also video interview segments with Mayo Clinic physicians.

The MayoClinic.com Health Decision Guides offer no community function. The site is developing its first Guide with tailored outcomes probabilities — on breast cancer adjuvant therapy — for release in late 2002.

#### Features

1. **Patient video interviews.** Multimedia patient stories are offered in the MayoClinic.com Health Decision Guides.
2. **Online community network.** No community or social support feature is offered in the MayoClinic.com Health Decision Guides.
3. **User-specific outcomes data.** No current MayoClinic.com Health Decision Guide offers tailored prognostic data, although one in development — on breast cancer adjuvant therapy — will offer that feature.
4. **Free, public access.** All MayoClinic.com Health Decision Guides are offered at no cost to the general public with no need to register.

### DIPEx

In 2001, DIPEx (Database of Individual Patient Experience), a new Internet multimedia resource was introduced. DIPEx is a not-for-profit organization based at the Department of Primary Care in the Institute of Health Sciences at the University of Oxford. As much as the NexCura tools emphasize the tailoring of data to the user, DIPEx emphasizes access to the experience of others who have faced the same decisions as the user [35]. The DIPEx Web site offers video clips, audio clips, and text transcripts of interviews with people describing their diagnoses, decisions, and experiences [36].

The site is not described as a decision-support tool. However, because it includes dozens of patient perspectives on how individual treatment decisions were made, because it includes evidence-based information, and because the patient perspectives are available free on the Web as a form of 24-hour support group, it may be an important tool for those making health care decisions.

There are more patient stories on this site than in any comparable site reviewed. (Users must have the Macromedia Flash™ and RealPlayer® plug-ins in order to use the multimedia on the site.) There are currently 4 programs on the site: on breast cancer, colorectal cancer, prostate cancer, and hypertension. Anyone can access the programs on the Web for free.

Each module has a "Forum" link, which is labeled as a place "where users can post messages, comments and exchange information with other members of the DIPEx community." However, visits to those parts of the Web site on August 6, 2002 were greeted with a message: "The DIPEx forums are currently undergoing redesign and evaluation."

#### Features

1. **Patient video interviews.** Multimedia patient stories are offered in the DIPEx modules — more than in any other Web site reviewed.
2. **Online community network.** DIPEx is currently redesigning and evaluating the concept of an online social support network whereby messages can be posted and information exchanged among users. That feature is not available as of August 6, 2002.
3. **User-specific outcomes data.** DIPEx modules do not offer tailored prognostic data.
4. **Free, public access.** All DIPEx modules are offered at no cost to the general public with no need to register.

## Discussion

### Potential for new tools

One of the more difficult decisions facing a woman with early-stage breast cancer regards adjuvant therapy — chemotherapy or hormone therapy or both — following surgical removal of the primary breast tumor. For reasons described below, women facing this decision may be prime candidates for an evaluation of a hybrid tool that uses each of the 4 key Web-enabled features reviewed in this paper. The decision is difficult because prognosis is uncertain and many women may feel there are significant trade-offs in their risk-benefit analysis. If the therapy helped all women but carried few side effects, the decision would be easier. A Web-based tool could individualize the information about the risks and benefits of adjuvant therapy. Such a tool could offer the woman the chance to hear from other women in videotaped interviews and in an online social support network. And, such a tool could be delivered free on the Web to the woman's home, where she can access the information in privacy, at her own pace, and repeatedly.

Among women with early-stage breast cancer, the chances of benefit from adjuvant therapy vary a great deal. For example, a woman with a primary tumor smaller than 1 centimeter and with no sign of spread to the lymph nodes is in a different risk category than a woman who has a 5-centimeter tumor and 10 positive lymph nodes. Yet, women in these 2 different risk categories may hear generally the same risk-benefit discussion about adjuvant therapy — in the clinical setting or in educational materials.

Developers of Web-based patient decision-support tools can use technology to improve the specificity of these messages. Sometimes women are given accurate, but very general statements about a treatment's possible benefits. Sometimes they may be given generic population-wide estimates (eg, "It is thought that adjuvant chemotherapy can provide a survival benefit at 10 years of 8-15%. This means that patients with invasive breast cancer who undergo 3-6 months of adjuvant chemotherapy or 5 years of hormonal therapy have an increase in survival of 8-15% compared to the patients who are just treated with surgery and or surgery and radiation therapy. Adjuvant chemotherapy helps 10-15% of the people who receive the therapy." [37]). But much more specific outcomes probabilities data could be given to a woman if she provided her age, the size of her primary tumor, and information about whether any cancer had been found in her axillary lymph nodes.

Mayo Clinic researchers have developed a beta-version of an online tool to deliver such tailored outcomes probabilities [38]. The tool has been described in the medical literature but has not been released to the general public [39].

The National Institutes of Health consensus panel on adjuvant therapy for breast cancer concluded its report in this way: "Methods to support shared decision-making between patients and their physicians have been successful in trials; they need to be tailored for diverse populations and should be tested for broader dissemination." [40]

There are many development and information delivery questions to test. Using the breast cancer adjuvant therapy example, it may seem intuitive that women would want to access information tailored to their own circumstances. But, there has not been adequate research conducted on the questions of whether women want to receive such tailored outcomes data, and of what difference it makes in their decision-making if they do receive such individualized information. The Mayo authors, studying this topic, believe the breast cancer adjuvant therapy decision demands such an offering. They wrote:

If anyone questions the uncertainties that abound regarding the prognosis of a primary breast cancer patient without, or with, various systemic adjuvant therapies, all one needs to do is ask four to five oncology colleagues to estimate 10-year disease free survival probabilities for a selected patient case. This will readily illustrate the wide variations of opinion in this area. To help physicians and patients make informed decisions, annual proportional risk reduction information needs to be translated into a more intuitive language [39].

Research has shown that many women with breast cancer do not recall receiving any estimates regarding prognosis [41].

Would it change decision-making if women did receive prognostic data? Would a woman over 50 years of age with a small primary tumor and no spread to the lymph nodes still choose adjuvant therapy if she knew that she had a 90% chance of being alive in 10 years without adjuvant therapy, and a 91% chance with standard chemotherapy? Would she endure the known side effects of chemotherapy to get that incremental benefit? Such questions have not been adequately studied.

Developers of Web-based decision-support tools should analyze whether women want such tailored outcomes data — and what they do with that information if they choose to receive it. Tools could be developed that offer women the chance to choose to see their own individualized prognostic data or — as an alternative — to receive valid, but generic information. The method and style of presentation of the outcomes data is an important consideration [42].

### Conclusions

Technology affords health communicators many new ways of reaching health care consumers using new media. The principal investigator of the CHESS project lists the following new directions interactive health communications should take over the next few years:

- conduct and disseminate more high-quality patient needs assessments;
- conduct more outcomes research on how interactive health communications systems work;
- explore the impact of developing systems that link patients directly to their providers;
- find ways to make it easy to use the Web, including encouraging sites to offer online user training [43].

Research begun at Dartmouth more than a decade ago — to track any possible post-viewing treatment shifts and actual treatment decisions made — is important to revive and continue. Tracking of patients' actual outcomes and experiences can help complete the cycle, with such data potentially being used to help guide new users' decisions.

Inadequate research has been done on the relative value of using some of the multimedia and community features of the Web in such decision-support tools. In the breast cancer example, it could be very helpful for the newly-diagnosed woman to be able to hear and watch video interviews with women who made different decisions that were rational in their respective cases. But, multimedia downloading or Web-streaming may tax many users' computer systems. The ability to add a community function, so that women could chat privately and anonymously with other women in a social support network, has been shown to have appeal. Web developers need to weigh the advantages and disadvantages of discussion groups moderated by a health care professional versus those that are not moderated. What do people trying to make health care decisions want and expect from such Web-based tools? Is the DIPEx site's emphasis on narrative storytelling any more or less important than NexCura's emphasis on delivering clinical trial information? Not enough research has been conducted or published to answer such questions. Several limitations of this review should be noted. In the fast-changing Internet environment, it is possible that a Web site that fit our inclusion criteria was overlooked.

It is also possible that some of the research questions raised herein have actually been addressed but have not been publicized because the information is held as the confidential information of for-profit ventures. However, this review demonstrates variation in the use of Web-enabled features as Internet decision-support tools are developed. This variation raises questions about what formative and process evaluation has been done and about what is being invested in the development of new tools.

In Web development, as in medicine, the old saying applies: "To a man with a new hammer, everything looks like a nail." It is possible that users do not find all of the Web's features helpful, desirable, or necessary.

It is possible to make available to many more people decision-support tools that contain all of the ideal features of prototypes of the past decade. Research can help ascertain user needs, what works best to address them, and how today's new media can be used most effectively. Then, perhaps, the hammer will have hit the nail on the head.

## Abbreviations

BPH: Benign Prostatic Hyperplasia
CHESS: Comprehensive Health Enhancement SupportSystem
DIPEx: Database of Individual Patient Experiences
FIMDM: Foundation for Informed Medical DecisionMaking
PDF: Portable Document Format
